# The Low-Abundance Plasma Proteome Reveals Differentially Abundant Proteins Associated with Breast Implant Capsular Contracture: A Pilot Study

**DOI:** 10.3390/proteomes12030022

**Published:** 2024-08-06

**Authors:** Md. Arifur Rahman, Ardeshir Amirkhani, Maria Mempin, Seong Beom Ahn, Anand K. Deva, Mark S. Baker, Karen Vickery, Honghua Hu

**Affiliations:** 1 Macquarie Medical School, Macquarie University, Sydney, NSW 2109, Australiacharlie.ahn@mq.edu.au (S.B.A.); anand.deva@mq.edu.au (A.K.D.); mark.baker@mq.edu.au (M.S.B.); kvickery@bigpond.net.au (K.V.); 2 Australian Proteome Analysis Facility, Sydney, NSW 2109, Australia; ardeshir.amirkhani@mq.edu.au; 3 Jinhua Institute of Zhejiang University, Jinhua 321016, China

**Keywords:** breast implants, capsular contracture, human plasma, Tandem Mass Tags, mass spectrometry, proteomics

## Abstract

Capsular contracture (CC) is one of the most common postoperative complications associated with breast implant-associated infections. The mechanisms that lead to CC remain poorly understood. Plasma is an ideal biospecimen for early proteomics biomarker discovery. However, as high-abundance proteins mask signals from low-abundance proteins, identifying novel or specific proteins as biomarkers for a particular disease has been hampered. Here, we employed depletion of high-abundance plasma proteins followed by Tandem Mass Tag (TMT)-based quantitative proteomics to compare 10 healthy control patients against 10 breast implant CC patients. A total of 450 proteins were identified from these samples. Among them, 16 proteins were significantly differentially expressed in which 5 proteins were upregulated and 11 downregulated in breast implant CC patients compared to healthy controls. Gene Ontology enrichment analysis revealed that proteins related to cell, cellular processes and catalytic activity were highest in the cellular component, biological process, and molecular function categories, respectively. Further, pathway analysis revealed that inflammatory responses, focal adhesion, platelet activation, and complement and coagulation cascades were enriched pathways. The differentially abundant proteins from TMT-based quantitative proteomics have the potential to provide important information for future mechanistic studies and in the development of breast implant CC biomarkers.

## 1. Introduction

Capsular contracture (CC) is a condition in which the collagen capsule around a breast implant hardens, tightens, and may cause pain and breast distortion [1]. Although the exact causes of CC are not fully understood, it is believed to be influenced by multiple factors [2]. Research suggests that subclinical infections and the formation of bacterial biofilms may play a role in the development of CC [2,3,4,5,6,7,8]. Studies using animal models have shown a link between biofilms and CC, particularly involving bacteria such as *S. aureus* and *S. epidermidis* [8]. Clinical studies showed a strong connection between biofilms and breast implant-related CC, as well as their role in chronic peri-implant inflammation [9,10,11,12]. Given the substantial evidence of the connection between biofilms and CC, there is an urgent need to identify the involvement of bacteria and/or host marker proteins in biofilm formation to facilitate early diagnosis and develop effective treatments.

Blood and its products (e.g., plasma or serum) are the most desirable source of biomarkers as they comprise released, cleaved, or secreted proteins and are readily accessible for proteomic analysis. Unfortunately, the widely varying composition and broad dynamic range of protein concentrations (greater than 12 orders of magnitude) pose tremendous challenges in the identification of clinically significant biomarkers against a backdrop of many higher-abundance proteins (e.g., albumin, IgG and transferrin) [13]. Eukaryotic translation elongation factor 1 alpha 1 (eEF1A1) has been identified as a potentially useful serum biomarker for the metastatic progression of human prostate cancer. However, further examination and validation is needed [1]. As higher-abundant proteins mask signals from low-abundance proteins, the identification of novel or specific proteins as biomarkers for a particular disease has been hampered, even with current high-resolution proteomic approaches. Depletion of higher-abundance proteins from human plasma/serum has been shown to advance the detection of relatively lower-abundance proteins [13,14,15].

Because of greater analytical characteristics, modern high-resolution mass spectrometry (MS)-based proteomics analysis is well-matched for fundamental human disease research and clinical diagnosis. Among the proteomic techniques used to identify biomarkers, the use of ‘Tandem Mass Tag’ (TMT) proteomics has the advantage of being able to achieve multiplexed quantification of up to ten samples simultaneously [16,17,18], with the ability to identify proteins between samples even in the lower abundance regions [19,20]. Additionally, it saves instrument time and reduces the complexity of experimental design [21].

Multiple studies have demonstrated differential regulation of proteins and identified novel/potential plasma biomarkers for a range of infectious diseases [14,22,23,24,25]. Further, human microfibril-associated protein-4 (MFAP-4) has been identified as a biomarker that can predict non-diseased liver versus cirrhosis with high diagnostic accuracy [26].

This study aimed to identify changes in protein expression in the plasma proteome of biofilm-related breast implant CC patients compared to those of healthy plasma samples using TMT-based MS analysis.

## 2. Materials and Methods

**Figure 1:**
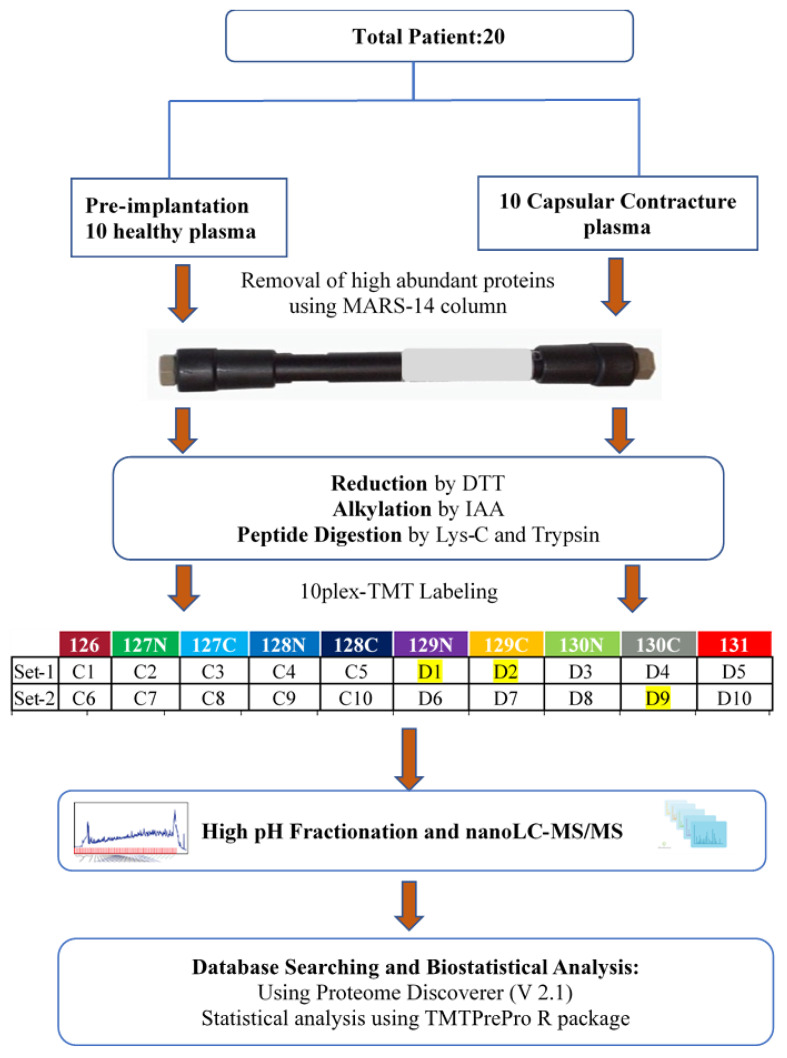
Experimental workflow for plasma depletion, TMT labelling, and analysis.

In the present study, a TMT-based high-resolution mass spectrometry approach was utilized to construct the plasma proteome (following depletion of high-abundant proteins) in healthy controls and patients with biofilm-related breast implant CC to explore disease-associated alterations of plasma proteins. The experimental workflow is illustrated in Figure 1.

### 2.1. Patients and Ethics

Human ethics approval was obtained from the Macquarie University Human Research Ethics Committee (Reference number 5201600427). After informed consent, patients were enrolled from various hospitals located in the eastern states of Australia. Blood was collected from 10 healthy patients receiving breast implants for the first time (control patients: C) and from 10 patients with symptomatic contracted capsules surrounding their breast implants (D) (Table 1). The mean age of the 10 control patients was 32 years, ranging from 25 to 39 years. The mean age of the 10 patients with CC was 50 ± 9 years, ranging from 37 to 64 years.

### 2.2. Collection and Processing of Human Plasma Samples

Up to 5 mL of blood was collected in ethylenediaminetetraacetic acid (EDTA) anti-coagulant tubes (BD Biosciences, Sydney, Australia) and plasma was separated by centrifugation at 800× *g* for 15 min at room temperature. Between 2 and 3 mL of plasma was removed and transferred in aliquots to 1.5 mL microcentrifuge tubes (Axygen; Fisher Biotech, Subiaco, WA, Australia) and centrifuged again at 1600× *g* for 10 min at RT to remove cellular debris. The plasma was then stored in 500 µL aliquots at −80 °C.

### 2.3. Depletion of High-Abundance Human Plasma Proteins

Plasma (40 µL) from each patient (10 healthy control, 10 capsular contracture) was processed for depletion of highly abundant proteins using the MARS-14 multiple affinity removal systems (Agilent Technologies, Santa Clara, CA, USA). As mentioned in the Agilent Technologies manual, “MARS-14 high capacity affinity column was specifically designed to employ anti-human plasma protein monoclonal antibodies to remove the 14 most abundant proteins (namely, human serum albumin, IgG, antitrypsin, IgA, transferrin, haptoglobin, fibrinogen, α2-macroglobulin, α1-acid glycoprotein, IgM, apolipoprotein AI, apolipoprotein AII, complement C3 and transthyretin) from human biological fluids such as plasma, serum, and cerebral spinal fluid (CSF)”. To deplete high-abundant plasma proteins, each sample was diluted 1:4 with buffer A and transferred to a 0.22 μm spin filter (Merck Millipore Ltd., Dublin, Ireland) and centrifuged for 2 min at 10,000× *g* and 4 °C to remove particulates. Depletion of each sample was performed on an Agilent 1260 HPLC system using MARS-14 column (4.6 × 100 mm) and proteins were eluted following the manufacturer’s instructions (Agilent Technologies). During each sample run, approximately 2 mL of flow-through fractions (at first low-abundant proteins were eluted, appeared between 11 and 15 min) were collected from each sample run followed by bound plasma proteins (high-abundant proteins). Each flow-through fraction containing low-abundant plasma protein samples was concentrated to approximately 200 µL using a 5 kDa MWCO membrane filter (Vivaspin, Sartorius, Goettingen, Germany) at 3200× *g* and 4 °C. Then, 2 mL of 1× PBS buffer was exchanged at the same conditions and concentrated to approximately 200 µL. A BCA protein assay was performed to determine the final low-abundant protein concentration.

### 2.4. Protein Reduction, Alkylation, and Digestion

Each sample containing 40 µg of protein underwent a series of processes including reduction, alkylation, and in-solution digestion, as described previously [27]. These enabled us to produce extremely complicated samples analyzed with TMT-based mass spectrometry. After reduction with 10 mM DTT and alkylation with 20 mM iodoacetamide IAA, the samples were digested using Lys-C and trypsin. Following acidification, centrifugation, and desalting, the samples were eluted, vacuum-dried, and stored at −20 °C.

### 2.5. TMT Labelling and High pH Fractionation

Reconstitute samples in 100 µL of 200 mM HEPES at pH 8.8, label with 20 µL of TMT for 1 h, add 8 µL of 5% Hydroxylamine to each sample, pool 3.2 µL of each, dry, reconstitute in 10 µL 0.1% formic acid and 2% ACN, centrifuge for 5 min at 14,000× *g*, then analyze by MS.

The data searching using Proteome Discoverer (V2.1), utilization of the normalization values, and high-pH RP-HPLC (Agilent Technologies) was performed as described previously [27]. The fractionated sample was pooled into 17 fractions, dried using a miVac (Genevac Ltd., Ipswich, England) concentrator, and reconstituted in an appropriate volume of 0.1% FA and 2% ACN for mass spectrometry analysis.

### 2.6. Nanoflow LC-ESI-MS/MS Using Q Exactive

Data were acquired using a Q Exactive mass spectrometer (Thermo Fisher Scientific, Dreieich, Germany) equipped with a nanospray source and an Easy nLC 1000 system (Thermo Fisher Scientific, Roskilde, Denmark), as described previously [27]. Briefly, each fraction, totalling 10 µL, was loaded onto a self-packed trap column 100 µm × 3.5 cm reversed-phase peptide trap with Halo^®^ 2.7 µm 160 Å ES-C18. The desalting process was performed with 20 µL of loading buffer [0.1% FA]. Then, the peptide trap was switched online with the analytical column followed by the peptides being eluted using linear gradients at a flow rate of 300 nL/min. Peptides were ionized by electrospray ionization, and data-dependent MS/MS acquisition was conducted using a Q Exactive. This consisted of one full MS1 scan acquisition (R = 70 K) from 350 to 1850 *m/z*, and ten HCD-type MS2 scans (R = 70 K).

### 2.7. Database Search, Statistical Analysis, and Bioinformatics

The raw data files were uploaded to Proteome Discoverer (version 2.1, Thermo Scientific). Subsequently, Sequest and Mascot were employed to analyse the data against all bacteria and human from the UniProt database. To avoid false-positive results, we set a high confidence criterion which includes master proteins, FDR < 1%, PSMs (high confidence), length > than 10 amino acids, and at least one unique peptide. Therefore, we can speculate that the bacterial proteins reported in this study meet acceptable criteria. For protein identification, the following parameters were used as described previously [27] “(i) peptide mass tolerance of 10 ppm; (ii) MS/MS tolerance of 0.1 Da; (iii) enzyme specificity set to trypsin with allowance for 1 missed cleavage; (iv) fixed modifications included carbamidomethyl (C), TMT10-plex (K), and TMT10-plex (N-term); (v) variable modifications encompassed oxidation (M), deamidation (N, Q), and acetylation (N-Terminus)”. Relative quantification was carried out dependent on the peak intensities of reporter ions in the MS/MS spectra. At least seven amino acids for peptide and greater than two peptides were essential. Below 1% FDR was selected as the cut-off for peptide identification. The quantification of proteins was based on the total intensity of the peptides allocated. After extracting protein ratios using Proteome Discoverer, additional processing and statistical analysis were performed using the TMTPrePro R package [28]. Metabolic pathways of identified proteins were assessed by utilising Kyoto Encyclopedia of Genes and Genomes (KEGG) mapper (https://www.genome.jp/kegg/tool/map_pathway2.html, accessed on 30 April 2019). The protein–protein interaction (PPI) network of significantly differentially abundant proteins was analysed by the Ingenuity Pathway Analysis (IPA) from Qiagen.

## 3. Results

### 3.1. Protein Identification

**Figure 2:**
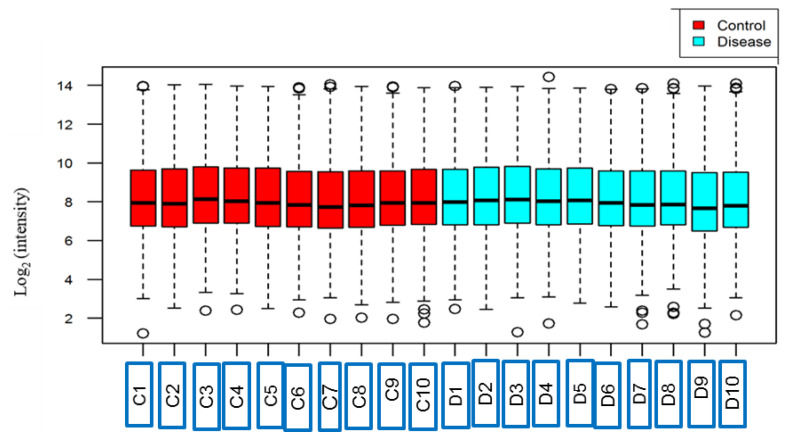
Box plots of log_2_ protein intensity average for each of the control samples (C1 to C10) and CC samples (D1 to D10).

A total of 581 proteins were identified in all 20 samples with a false discovery rate (FDR) < 1%. The 10-plex data set 1 identified 539 proteins (in 10 samples) while 492 proteins were identified in data set 2 (in another 10 samples). Of these, 450 non-redundant proteins were commonly identified in all 20 samples. The full list of raw human plasma proteome data of breast implant capsular contracture patients and healthy controls is available in the Supplemental Table S1. Box plot evaluation revealed that the protein intensity medians of all 20 samples were very similar, implying no bias towards any sample or data set (Figure 2). Additionally, in our data, we identified four bacterial proteins with a high level of confidence (Table 2). To improve the accuracy of identifying bacterial proteins and minimize false-positive results, we established stringent criteria including master proteins, FDR 10 amino acids, and at least one unique peptide. Therefore, we can speculate that the bacterial proteins reported in this study meet acceptable criteria. It was demonstrated that biofilm infection of breast implants is multi-species [11]. However, bacterial proteins may occur in healthy control patients as low-grade bacteremia occurs during teeth cleaning.

Filtering the TMT data set using criteria of *p*-value ≤ 0.05, we identified a total of 43 proteins, in which 16 proteins were significantly differentially expressed (fold change > 1.2), among them 5 proteins were upregulated whereas 11 proteins were downregulated in the CC group compared with the control group. A full list of all significantly differentially abundant proteins is provided in Table 3. The low number of differentially regulated proteins between patients with contracture and the healthy controls is likely due to the nature of breast implant-related contracture, in that these patients are systemically healthy like control patients. Patients with breast implant contracture are generally receiving treatment for aesthetic reasons, not health/disease-related reasons.

**Figure 3:**
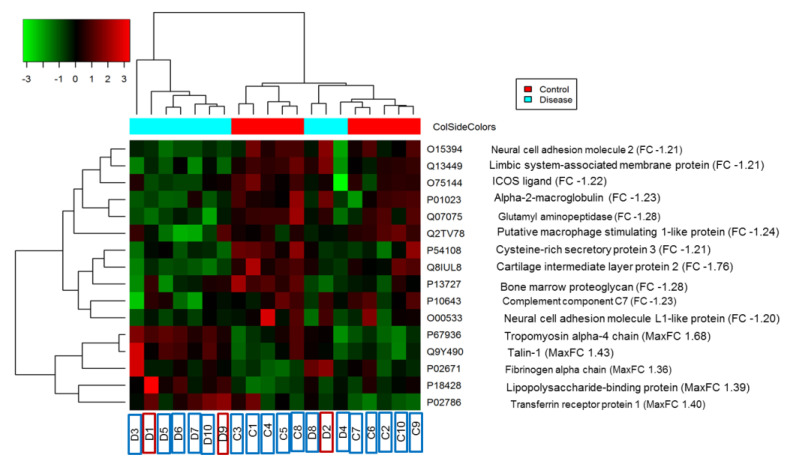
Heatmap analysis of the significantly changed proteins. Rows denote proteins and columns denote different samples. Each cell’s colour represents a shift in protein expression; red indicates increased and green indicates decreased relative to the healthy controls. In this study, we performed two sets of TMT-based analysis and combined the two sets for analysis using the TMT-PrePro R package.

A heatmap analysis of 16 differentially abundant proteins using a hierarchical clustering and correlation distance metric was generated to illustrate the alteration of expression levels in the CC group versus the control group (Figure 3). As shown in the dendrogram, an increasing red colour indicates increasing levels of protein expression (Figure 3). Therefore, in the region where these protein peaks are shown in red, the most notable area of upregulation occurred in the disease group.

### 3.2. Gene Ontology Analysis of Identified Proteins

**Figure 4:**
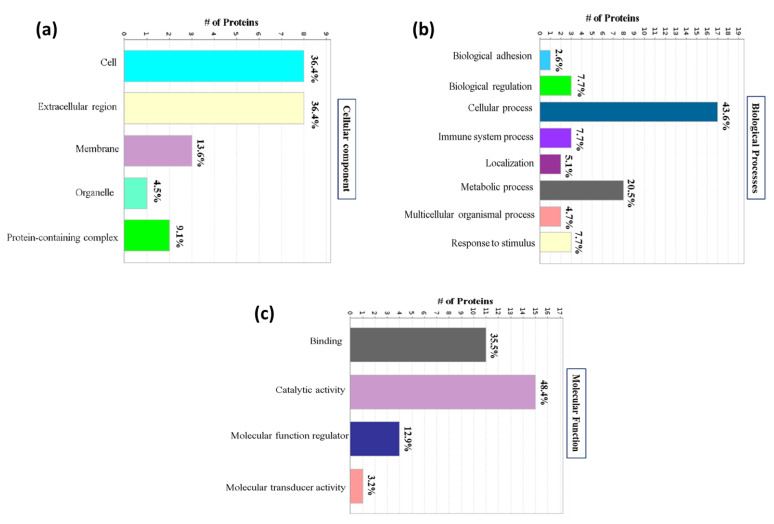
Classification of significantly identified proteins based on their functional annotations using GO. (**a**) Cellular component category; (**b**) biological processes category; (**c**) molecular function category.

To further understand the functions of the significantly altered proteins, the cellular component, molecular function, and biological process of the 43 proteins were explored using Gene Ontology (GO) annotation with PANTHER. In the cellular component category of GO, most of the proteins had a cellular (36%) and extracellular location (36%), followed by a membrane location (14%) (Figure 4a).

According to the analysis of biological processes, the primary functions were cellular processes (44%) and metabolic processes (21%); while others were related to biological regulation (8%), immune system processes (8%), response to stimulus (8%), and so on (Figure 4b).

In the molecular function category of GO, the top two molecular function terms were catalytic activity (48%) and binding (36%), followed by molecular function regulator (13%) and molecular transducer activity (3%) (Figure 4c).

### 3.3. KEGG Pathway and Protein–Protein Interaction Analysis of the Identified Proteins

We analyzed the TMT results using KEGG pathways to identify pathways affected by CC compared to control patients. Among the 16 significantly differentially abundant proteins, 11 proteins were identified in recognised pathways. The 11 unique differentially regulated proteins were mainly involved in platelet activation, focal adhesion formation, hematopoietic cell lineages, endocytosis, phagosome, ferroptosis, NF-kappa B signalling pathway, toll-like receptor signalling pathway, complement/coagulation cascades, and cell adhesion molecules (CAMs) (Table 2). Further detailed analyses of the roles of the significantly differentially abundant proteins were conducted using Qiagen’s Ingenuity Pathway Analysis (IPA) and by searching the current literature. 

**Figure 5:**
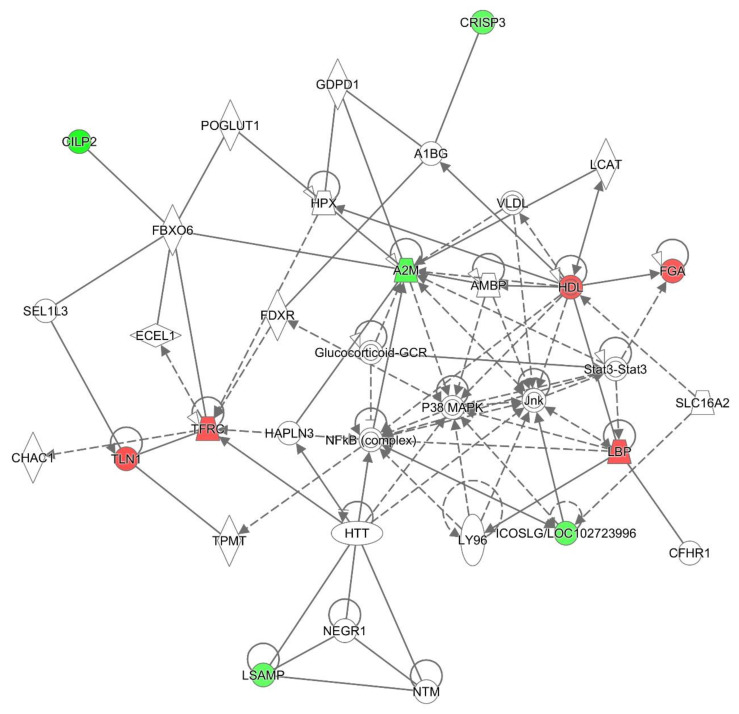
PPI network analysis of the significantly differentially regulated proteins in the CC group compared to the healthy group using IPA. Interactions between two proteins are indicated with grey lines. Red nodes denote upregulated proteins and green nodes denote downregulated proteins.

Protein–protein interaction (PPI) analysis of 16 differentially abundant proteins showed a relatively complex network with several distinct biological subgroups that contained highly connected proteins. As depicted in Figure 5, proteins involved in platelet activation, complement/coagulation cascades, immune response, inflammatory response, and cell adhesion molecules (CAMs) were highly connected, indicating that the functional network of these processes contributes to biofilm-related breast implant CC pathophysiology.

## 4. Discussion

Among breast implant-associated complications, CC is the most common postoperative complication observed worldwide. The mechanisms that lead to CC remain unclear. There is currently no effective technique for the early identification of biofilms and eradication of implant-associated biofilm infection that often requires painful and expensive removal or replacement of contaminated devices. Given the vast amount of evidence for the significant correlation between biofilm and CC [29], potential biomarkers may provide a clue of the existence of bacterial pathogens in plasma and/or host marker proteins in biofilm formation. These might be used to develop early-stage diagnostic and/or new therapeutic interventions.

We used a TMT-based high-resolution proteomic strategy to identify and quantify the differentially abundant proteins/proteoforms in the human plasma proteome caused by biofilm-related breast implant CC. Labeling-based proteomic techniques, such as TMT-based MS, allow for a more comprehensive analysis of the complexity of human proteomes and are becoming increasingly popular for identifying plasma biomarkers for diseases like cancer, sepsis, and infectious diseases [30,31,32,33].

Acute phase response associated high-abundance proteins such as C-reactive protein are changed in numerous disease states like myocardial infarction, trauma, and rheumatologic diseases [34,35]. In addition, the wide dynamic range of protein concentrations in plasma samples and the depletion of high-abundance plasma proteins improve the detection of relatively lower-abundance proteins which might be clinically important [23]. Therefore, we first performed depletion of high-abundance proteins (such as albumin and IgG) using the immune affinity-based depletion method (MARS-14) to improve the depth of detection in plasma samples followed by TMT-based MS. This step is crucial to increase proteomic coverage and detect clinically significant proteins/proteoforms in the complexity of human proteomes. As a result of combining depletion with TMT labelling, a total of 450 proteins were identified among both CC and healthy groups. Sixteen significantly differentially abundant proteins were identified in the biofilm-related breast implant CC compared with healthy controls. Three samples obtained from patients with contracture were clustered with the control samples. This is probably a reflection of the subclinical progression of the disease. 

We identified five significantly upregulated plasma proteins (i.e., tropomyosin alpha-4, talin-1, transferrin receptor protein 1, lipopolysaccharide-binding and fibrinogen alpha chain) and 11 downregulated plasma proteins (i.e., cartilage intermediate layer protein 2, alpha-2-macroglobulin, bone marrow proteoglycan, complement component C7, ICOS ligand, neural cell adhesion molecule 2) as potential biomarkers.

Tropomyosin belongs to a multi-isoform family, expressed in muscle and non-muscle cells including fibroblasts, epithelial cells, and platelets. In muscle cells, tropomyosin plays a crucial role in muscle contraction by regulating actin–myosin binding in response to calcium ion flux. More research is required to know the deeper function of tropomyosin in non-muscle cells; however, tropomyosin is an essential component of the cytoskeleton and contributes to cell contraction regulation [36]. Multiple studies report the upregulation of tropomyosin alpha chain in diseases such as oesophagal carcinogenesis, liver cirrhosis, Behçet’s disease, and hepatocellular carcinoma [26,36,37,38]. In addition, autoimmune responses to tropomyosin have been observed in other chronic diseases, including ulcerative colitis [39], Alzheimer’s disease [40], and autoimmune hepatitis [41]. A proteomic study showed significant upregulation of tropomyosin alpha-4 in oesophageal squamous cell carcinoma and fibroblasts adjacent to cancer cells expressed high levels of tropomyosin alpha 4 chain [37]. This suggested that, in addition to the contribution of tropomyosin alpha 4 chain to morphological change and the cytoskeleton stability of cancer cells, it may be associated with infiltration or metastatic ability [37]. Another interesting proteomic study using serum of liver cirrhosis patients found significant upregulation of tropomyosin alpha 4 chain, which is probably synthesized by “myofibroblast-like” triggered hepatic stellate cells, and linked with actin filaments of myofibrils and stress fibres [26]. They also suggested that these are sources of hepatocellular necrosis-associated mediators, such as extracellular secretion of free radicals, also referred to as oxidant stress, intracellular components, and signalling molecules, and that these mediators may be circulating, intercellular, or acting on the same cell [26]. Free radical (oxidant stress)-mediated necrosis may weaken stellate cell stimulation with increased synthesis of smooth muscle proteins. The tropomyosin alpha 4 chain can be observed in serum/plasma with a significant range from patients with liver cirrhosis and other cirrhosis-associated conditions [26]. In addition, there is an increase in the cytoskeleton with increased fibrosis.

**Figure 6:**
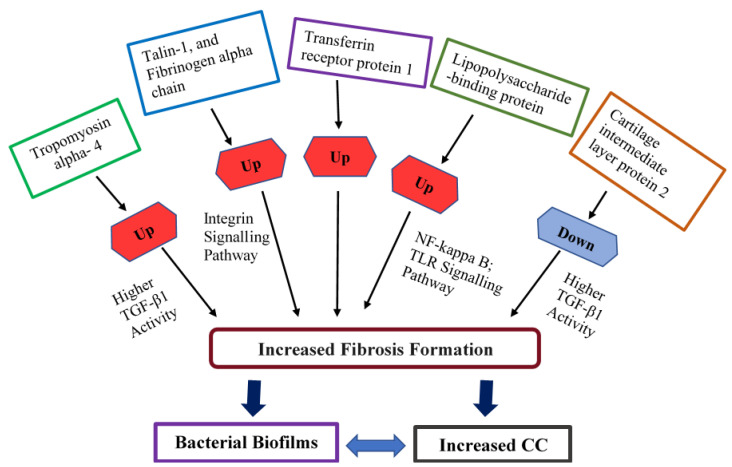
Potential target regulators and associated pathways demonstration leading to CC and a possible link with bacterial biofilms.

Interestingly, higher numbers of various types of fibroblasts, including myofibroblasts, are observed in CC [42,43]. Transforming growth factor beta-1 (TGF-β1) and stress-associated responses play a significant role in the regulation of these molecular factors as well as linked with the repair of wounds [42]. There is a positive correlation between bacterial biofilms and TGF-β1 expression, which is consistent with myofibroblast activity and fibrosis [44]. Myofibroblasts can produce collagen and a specific form of fibronectin [45] and may contribute to the formation of a contracted capsule around a breast implant [46]. The excessive collagen and fibronectin can be exploited by bacteria which facilitates their adhesion to extracellular matrix molecules [47,48,49,50]. Bacteria multiply and form biofilms once they are attached. The higher myofibroblast activity and count may lead to increased tropomyosin alpha 4 chain production, potentially causing elevated fibrosis and connective tissue contraction, possibly associated with bacterial biofilms. It is not entirely surprising that the tropomyosin alpha 4 chain can be observed in higher levels in plasma from patients with biofilm-related breast implant CC (Figure 6).

Talin-1 (TLN1), located at the adhesion complex between cells and their extracellular matrix (ECM), regulates integrin and focal adhesion signalling and is expressed mainly in the liver, kidney, stomach, spleen, lung, and vascular smooth muscle. Multiple studies have reported that overexpression of talin-1 can promote prostate cancer cell adhesion, migration, and invasion [51,52,53]. Further studies also reported the upregulation of talin-1 in inflammatory diseases [54]. The upregulation of talin-1 in the cholangiocarcinoma group, which is associated with the ECM, cell mobility, and adhesion, was reported to play vital roles in ECM remodelling, and attachment and metastasis were known to happen during human cholangiocarcinoma [55]. 

Another upregulated protein, fibrinogen alpha chain, is involved in platelet activation, complement and coagulation cascades, and (bacterial) infection/inflammation mechanisms. This is especially so in conditions such as vascular wall disease, periprosthetic joint infection, and lung and kidney fibrosis [56,57,58]. Fibrinogen, the precursor of fibrin is a soluble glycoprotein, comprised of three polypeptide chains called Aα, Bβ, and γ, and these play a vital function in triggering and facilitating inflammation, where higher amounts of fibrinogen alpha adhere to the surface of breast implants [59]. On the other hand, ECM proteins (e.g., fibronectin and fibrinogen) act as ligands to bind cell surface integrin receptors, and the capability of fibronectin to encourage biofilm development is related to the fibrinogen-binding A domain, which facilitates cell to cell attachment via homophilic bonds of low-affinity [58,59]. If bacteria (e.g., *S. aureus*), in some cases, may not come into contact directly with the cell, its FnBPs (e.g., fibronectin and fibrinogen) ease binding to host plasma proteins. These proteins can function as linking molecules between the bacteria and the receptors of a host cell. FnBPs can cause internalisation via integrin receptors, and internalisation can lead to the persistence of infection intracellularly [60].

This study found higher levels of TLN1 and fibrinogen alpha chain in the plasma of patients with biofilm-related breast implant capsular contracture compared to healthy controls. This suggests that TLN1 and fibrinogen alpha chain might be novel candidates of biofilm-related breast implant CC for the diagnosis of breast implant CC (Figure 6). In addition, since the integrin signalling pathway plays a vital role in the pathogenesis of inflammation and the close association of the integrin signalling pathway with TLN1 and fibrinogen alpha chain, we assume that the integrin signalling pathway could be involved in the pathogenesis of breast implant CC (Figure 6).

Transferrin receptor protein 1 (TFRC), the main receptor for cellular iron uptake into cells, is ubiquitously expressed in all tissues and plays a significant role in carrying iron to the host cell during infection and inflammation [61,62,63]. For instance, upregulation of transferrin receptor protein 1 has been observed in animal models of inflammation [64,65] and in patients with acute respiratory distress syndrome [65]. Further, the upregulation of transferrin receptor protein 1 may decrease the accessibility of vital iron to cause bacterial invasion and is likely to be useful to the host during acute inflammation. Iron deposition in tissues is connected with toxicity in chronic inflammation [66] and initiates the development of fibrosis and liver disease in the final stages, for example, liver disease or chronic hepatitis C [63]. 

Another upregulated protein identified was lipopolysaccharide-binding protein (LBP), which relates to multiple pathways such as the nuclear factor (NF)-kappa B signalling pathway and toll-like receptor signalling. LBP is an acute-phase protein that is abundantly synthesised in hepatocytes, responsible for the binding to the lipid A segments of lipopolysaccharide (LPS), and brings on toll-like receptor-4 (TLR-4), cluster of differentiation 14 (CD14), and other signalling pathways in inflammatory responses [67,68,69]. Several studies have reported that plasma/serum levels of LBP are significantly elevated in patients with conditions associated with systemic inflammatory responses, sepsis, as well as acute and chronic infections [70,71,72,73,74]. These studies indicate that LBP might be useful as a potential biomarker for inflammatory reactions from both infectious and non-infectious backgrounds (Figure 6).

Among the significantly downregulated proteins, cartilage intermediate layer protein 2 (CILP2) is an extracellular carbohydrate protein that exists in the ECM and is expressed in skin, heart, articular cartilage, adipose tissue, plasma, etc. The mentioned tissues contain various matrix-binding molecules, including collagen types I, III, V, and VI. Reduced CILP expression is significantly correlated with TGF-β1 activity. In a prior study with obese men, plasma samples indicated decreased CILP levels, potentially associated with increased TGF-β1 activity and inhibition of adipogenesis [75]. In the above study, they observed higher TGF-β1 activity secreted by human adipose tissue in obesity, which also showed a similar trend with myofibroblast activity and fibrosis and may be linked with the development of CC around an implant (Figure 6).

In this preliminary study, we analysed a small group of patients to find blood plasma protein markers related to capsular contracture. While we gained valuable insights, the limited sample size makes it difficult to draw firm conclusions. Future research should involve a larger and balanced patient group to validate the identified biomarkers. Future studies should incorporate validation steps, such as ELISA or immunoblotting with specific antibodies, to confirm these findings and provide stronger evidence for the identified biomarkers’ relevance and potential clinical utility. Despite this limitation, the study has provided a series of potential targets for future research. 

Age discrepancies between the control group and CC patients were due to the nature of the study design. The control patients were those who received the breast implants for the first time and the CC patients were when they faced any breast implant-associated complications. However, CC is a chronic disease that takes years to become clinically evident. Age-related changes could account for some observed differences, confounding the study’s results. Future studies should aim to include age-matched controls to address this issue and better isolate the biomarkers specific to CC. 

This study showed how biofilm-related breast implant CC affects the human plasma proteome. By identifying specific protein/proteoforms and their associated pathways, researchers can better understand the mechanisms behind CC and develop more effective diagnostic and therapeutic strategies.

## 5. Conclusions

This study used a quantitative proteomic approach to compare the proteome of breast implant patients with biofilm-related complications to healthy controls. These complications trigger a non-specific inflammatory response. The identified differentially abundant proteins in CC patients provide new insights into biofilm-related breast implant infections. Further research on the pathways involved in these proteins is needed to understand the underlying mechanisms.

## Figures and Tables

**Table 1:** Details of selected patients used in this study.

**SN**	**Patient ID**	**Age (Years)**	
1	C1	33	
2	C2	34	
3	C3	25	
4	C4	31	
5	C5	29	
6	C6	39	
7	C7	36	
8	C8	35	
9	C9	29	
10	C10	32	
**SN**	**Patient ID**	**Age at Diagnosis (Years)**	**Bacteria Isolated**
1	D1	42	*Enterococcus* spp., *S. epidermidis*
2	D2	58	*S. epidermidis*
3	D3	62	N/A
4	D4	39	*Micrococcus luteus*
5	D5	49	N/A
6	D6	64	*S. epidermidis*
7	D7	49	N/A
8	D8	37	*S. aureus*
9	D9	44	*S. epidermidis*
10	D10	52	N/A

N/A: no bacteria isolated.

**Table 2:** List of bacterial proteins found in this study.

Accession	Description	Fold Change	Subcellular Location	KEGG Pathway
P15636	Protease 1 OS = *Achromobacter lyticus*	1.0	Secreted	N/A
P06654	Immunoglobulin G-binding protein G OS = Streptococcus sp. group G, GN = spg	1.1	Cell Wall	N/A
Q890U2	Glutamine--fructose-6-phosphate aminotransferase [isomerizing] OS = Clostridium tetani (strain Massachusetts/E88), GN = glmS	1.3	Cytoplasm	Alanine, aspartate and glutamate metabolism, Biosynthesis of antibiotics, Metabolic pathways, Amino sugar and nucleotide sugar metabolism
A9MPX1	Zinc transporter ZupT OS = Salmonella arizonae (strain ATCC BAA-731/CDC346-86/RSK2980), GN = zupT	1.0		N/A

N/A: not available.

**Table 3:** Functional classification of the significantly differentially abundant proteins in the CC groups compared with the control groups (*p* < 0.05).

Accession ID	Protein Name	Mean Disease /Mean Control	Fold Change	Protein Pathways	Subcellular Localization
Q8IUL8	Cartilage intermediate layer protein 2	0.579	−1.7		Secreted
P67936	Tropomyosin alpha-4 chain	1.687	1.7	Hypertrophic cardiomyopathy, Adrenergic signaling in cardiomyocytes, Dilated cardiomyopathy (DCM), Cardiac muscle contraction	Cytoskeleton /Cytoplasm
Q9Y490	Talin-1 OS = Homo sapiens	1.440	1.4	Rap1 signaling pathway, Human T-cell leukemia virus 1 infection, Platelet activation, Focal adhesion	Cytoskeleton /plasma membrane
P02786	Transferrin receptor protein 1	1.399	1.4	Hematopoietic cell lineage, Endocytosis, HIF-1 signaling pathway, Phagosome, Ferroptosis	Plasma membrane /secreted
P18428	Lipopolysaccharide-binding protein	1.389	1.4	NF-kappa B signaling pathway, Tuberculosis, Toll-like receptor signaling pathway, Salmonella infection	Secreted
P02671	Fibrinogen alpha chain	1.360	1.4	Platelet activation Complement and coagulation cascades	Secreted
P01023	Alpha-2-macroglobulin	0.758	−1.3	Complement and coagulation cascades	Secreted
P13727	Bone marrow proteoglycan	0.780	−1.3	Asthma	Secreted
Q07075	Glutamyl aminopeptidase	0.781	−1.3	Renin–angiotensin system	Plasma membrane
Q2TV78	Putative macrophage stimulating 1-like protein	0.802	−1.2		Secreted
P10643	Complement component C7	0.813	−1.2	Prion diseases, Complement and coagulation cascades, Systemic lupus erythematosus	Secreted
O75144	ICOS ligand	0.814	−1.2	Cell adhesion molecules (CAMs), Intestinal immune network for IgA production	Plasma membrane
O15394	Neural cell adhesion molecule 2	0.821	−1.2	Cell adhesion molecules (CAMs), Prion diseases	Plasma membrane
Q13449	Limbic system-associated membrane protein	0.823	−1.2		Plasma membrane
P54108	Cysteine-rich secretory protein 3	0.824	−1.2		Secreted
O00533	Neural cell adhesion molecule L1-like protein	0.827	−1.2		Plasma membrane

## Data Availability

Data are available via ProteomeXchange [76] with the identifier PXD044649.

## Supplementary Materials

The following supporting information can be downloaded at: https://www.mdpi.com/article/10.3390/proteomes12030022/s1, Supplementary Table S1. Raw human plasma proteome data of breast implant capsular contracture patients and healthy controls.
